# Quantification and Influence of IL-1β on Pain and Inflammatory Response after Placement of a Cement–Screw-Retained Restoration

**DOI:** 10.3390/jcm13061669

**Published:** 2024-03-14

**Authors:** Lady Arbelaez-Bonozo, Serafín Maza-Solano, María Baus-Domínguez, Raquel Gómez-Díaz, Gonzalo Ruiz-de-Leon-Pacheco, Daniel Torres-Lagares, María-Angeles Serrera-Figallo

**Affiliations:** 1 Department of Stomatology, Faculty of Dentistry, University of Seville, 41009 Seville, Spain; ladarbbon@alum.us.es (L.A.-B.); serafinmazasolano@gmail.com (S.M.-S.); danieltl@us.es (D.T.-L.); 2 Institute of Biomedicine of Seville, 41013 Seville, Spain; rgomez-ibis@us.es; 3 Gregorio Marañón General University Hospital, 28007 Madrid, Spain; ruizdeleong@gmail.com

**Keywords:** dental implant abutment design, CAD-CAM, peri-implant crevicular fluid, interleukin-1β, dental implants

## Abstract

**Background:** The objective of this study was to evaluate the pain and inflammatory response in soft tissues using healing and prosthetic abutments of different diameters and lengths. **Methods:** The study population was rehabilitated with Astra Tech EV single implants (Dentsply Sirona, Atlantis, Dentsply Sirona S.A., Barcelona, Spain) of 4.2 and 4.8 millimetres in diameter in the upper and lower maxilla and loaded with custom abutments digitally designed using Dentsply Sirona’s Virtual Atlantis Design software (Atlantis WebOrder, Dentsply Sirona S.A., Barcelona, Spain), version 4.6.5. The custom abutments had a larger diameter than the healing abutments to evaluate for biomarkers through ELISA. **Results:** Rehabilitations in the mandible and with healing abutments with diameters less than 4.29 mm and rehabilitators with diameters less than 2.18 mm elicited a higher pain and inflammatory response and, in turn, higher interleukin-1β values. **Conclusions:** Greater inflammation was evident in cases in which healing abutments with reduced diameter were used compared to the same subsequent rehabilitation with prosthetic abutments with larger diameters.

## 1. Introduction

The influence of the formation of supracrestal insertion tissue around implants is frequently studied because peri-implant soft tissues provide hygiene, aesthetics, and health to a prosthesis or crown on implants, forming an aesthetic biological contour [1].

The dimensions of peri-implant soft tissues were described by Tomasi and collaborators based on the analysis of human biopsies [2]. In 2013, Tomasi et al. described soft tissue dimensions of around 3.6 mm, while other authors claim an average of 4.20 mm [3], including a barrier epithelium of 1.9 mm and connective tissue of 1.7 mm.

It has been shown that the portion of the mucosa that is in intimate contact with the prosthetic abutment surface can be divided into two distinct zones: a marginal zone that harbours a junctional epithelium and a more apical zone that is composed of fibre-rich connective tissue [4,5,6].

These areas may vary depending on the cervical design, gingival biotype, and implant depth.

From in vitro [7] and in vivo [8] experiments, it has been concluded that the junctional epithelium of the peri-implant mucosa through hemidesmosomes is adherent to the titanium surface, while other studies on dogs [4,9] have suggested that the connective tissue in the interface zone has the characteristics of scar tissue (sparse in cells and vascular structures but rich in collagen fibres), which is firmly attached to the abutment.

Subsequent studies have suggested that the vertical mucosal thickness necessary for establishing the correct biological width around dental implants should be at least 2 mm to avoid marginal bone loss [10,11]. It should be emphasised that the minimum and maximum thickness values serve as a starting point, when in fact what we are looking for is to give the patient back what they had, i.e., if a patient has a thin biotype where their gingiva measures 2.5 or 3 mm, it is necessary to make the measurement and provide that gingival space at the time of acquiring a prosthetic abutment or when making the gingival contour of a provisional restoration.

It has been observed that it is possible to prevent peri-implant bone remodelling if we adapt the vertical position of the implant to the thickness of the soft tissue. Based on this principle, the implant should be submerged in the gingival margin between 3 and 4 mm depending on the size of the implant [12].

This study is significant because of the importance of the biological aesthetic of the surrounding shape in single-tooth restorations; the technique used will depend on the clinical approach of the case, the immediate or delayed placement of the implant, and the need to improve the adjacent soft tissues. Studies by different authors have shown the influence of inflammation, which can cause highly localised destruction of connective tissues and stimulate epithelial proliferation [13]. Predisposing factors are the shortage of space in the peri-implant tissues, the use of inadequate attachments, and poor patient hygiene, among others, which can lead to gingival recession, exposure of attachments, and implant components; therefore, in response to inflammatory agents, infections, or microbial endotoxins, a dramatic increase in IL-1β production by macrophages and other types of cells is observed [14]. IL-1β plays a central role in immune and inflammatory responses [15].

In particular, in this study, we evaluated the use of healing abutments with variable diameters and lengths to determine the suitable measurements. This will help us make appropriate decisions when performing second-stage surgery and use attachments that favour the maintenance of hard and soft tissues in the long term.

An analysis of variables based on the diameter and height of the abutments was conducted. In this study, it was possible to observe the pain and inflammatory response in the maxillary and mandibular post-surgery with single-unit rehabilitations on implants whose healing screw diameter was smaller than the diameter of the rehabilitating abutment.

The null hypothesis was that the greater the amount of Il-1β expected, the greater the difference in diameter and height between the prosthetic abutment and the healing cap as a consequence of the greater pain and inflammatory response.

## 2. Materials and Methods

### 2.1. Type of Study

The prospective observational cohort study was approved by the Andalusian Biomedical Research Ethics Coordinating Committee (Code US-DTL-2022.1) and complies with all the guidelines of the World Medical Association Declaration of Helsinki: Ethical Principles for Medical Research Involving Human Subjects [16].

This is an observational study whose invasive procedure was the collection of saliva samples from the peri-implant tissues of implants rehabilitated with different diameters of healing and prosthetic abutments.

All patients signed an informed consent form based on this study and understood and accepted the type of treatment carried out on them.

### 2.2. Patient Selection

A series of patients meeting the following inclusion and exclusion criteria were selected:

Inclusion criteria:

- Adult patients over 40 years of age;
- Edentulous patients requiring single crowns;
- Single edentulous spaces 6 to 8 mm in height.

Exclusion criteria:

- Patients with uncontrolled chronic diseases;
- Patients with immune diseases;
- Patients with smoking habits, alcoholism, or narcotic drug use;
- Patients medicated with steroids or bisphosphonates;
- Patients with active periodontal disease;
- Patients with poor hygiene habits.

### 2.3. Surgical Procedure

Patients were surgically treated with implants (Astra Tech EV, Dentsply Sirona S.A., Barcelona, Spain) of 4.2 mm or 4.8 mm diameter in the upper and lower jaws.

The implants received healing abutments and were subsequently connected with customised abutments (Atlantis, Dentsply Sirona S.A., Barcelona, Spain), which were digitally designed with the Virtual Atlantis Design (VAD) software (Atlantis WebOrder, Dentsply Sirona S.A., Barcelona, Spain), version 4.6.5, which provided the necessary precision and a combination of biological, anatomical, and engineering parameters, providing beneficial conditions for soft tissue healing and adaptation of the final restoration.

All prosthetic abutments were individualised with a larger diameter than the healing abutment in order to evaluate the pain and inflammatory response of patients after definitive rehabilitation, which was quantified by the presence of biomarkers present in the crevicular fluid, such as interleukin-1 beta (IL-1β) proteins, in an ELISA test.

### 2.4. Prosthetic Procedure

The material of choice for the preparation of customised abutments was zirconium oxide (InCoris ZI meso, Dentsply Sirona, DeguDent GmbH Rodenbacher Chaussee Hanau-Wolfgang, Hanau, Germany) for CAD/CAM production. The same abutment design was used for all patients (customised Atlantis-type titanium nitride abutments from Dentsply Sirona). The only variant was the measurement of the available soft tissues and, according to that, the surface to make contact with the critical and subcritical profiles, what we now know as the B, C, and E zones [17].

For the prosthetic crown, zirconium oxide discs were chosen (Cercon XT ML, Dentsply Sirona, DeguDent GmbH Rodenbacher Chaussee Hanau-Wolfgang, Hanau, Germany), which have 750 MPa of resistance to bending over their entire length, taking the precaution of maintaining a minimum thickness of restoration of 1.5 mm as a margin of error and the advantage of having a 49% translucency in degradation throughout the restoration, obtaining a more aesthetic result.

The cases were sent to the dental laboratory, which continued with the milling process of the single-unit restorations on implants.

The cases were planned to be of the indirect cement–screw type. Dual-curing self-adhesive resin cement (Relyx unicem 2, 3M ESPE AG, Dental Products Seefeld, Seefeld, Germany) was used because of its dimensional stability and high resistance to microfiltration due to the long-term dissolution of the material.

Finally, the single crowns were installed in each of the patients, and the pain scale measurement was performed immediately.

### 2.5. Pain and Inflammation Scales

These were manually annotated on paper with a template for each patient, which consisted of variables from 0 to 5, for greater patient comprehension, with 0 being no pain and 5 being maximum pain. Specifically, value 0 was assigned to patients who had no pain at any time during the placement or afterwards, value 1 to patients whose pain did not last more than 10 min, value 2 to patients whose pain disappeared before 24 h, value 3 to patients whose pain disappeared between 24 and 48 h after placement, value 4 to patients whose pain disappeared after 72 h, and value 5 to patients whose pain lasted for more than 72 h.

The inflammation scale was also evaluated, also consisting of variables from 0 to 5, and scored manually on paper with a template for each patient, with 0 being no inflammation and 5 being maximum inflammation. Specifically, value 0 was assigned to patients who had no inflammation at any time during or after placement, value 1 to patients whose inflammation did not last more than 10 min, value 2 to patients whose inflammation disappeared within 24 h, value 3 to patients whose inflammation disappeared between 24 and 48 h after placement, value 4 to patients whose inflammation disappeared within 72 h, and value 5 to patients whose inflammation lasted for more than 72 h.

### 2.6. Sampling

Sample collection was carried out 4 h after the delivery of the rehabilitation to each patient.

The collection of inflammation biomarker samples was taken at 4 sites (mesiobuccal, distobuccal, mesiolingual, and distolingual) of each implant. To avoid contaminating the sample with saliva, a sterile paper collection strip (PerioPaper strips, Oralflow, Smithtown, NY, USA) was inserted into the peri-implant sulcus for 30 s according to the manufacturer’s instructions.

The four strips from each implant were pooled in Eppendorf centrifuge tubes and subsequently stored at −80 °C until further processing.

### 2.7. Biomarker Analysis Using ELISA Technique for IL-1β

The kit used for the ELISA technique was the KIT Quantikine^®^ HS ELISA Human IL-1β/IL-1F2 Immunoassay (Ref. HSLB00D). It is a sandwich-type enzyme immunoassay technique in which the plate is treated with a monoclonal antibody specific for human IL-1β. In the presence of IL-1β in the sample, the antibody fixed to it will bind to the plate. On the day of processing, they were thawed and 400 µL of Calibrator Diluent RD5T were added. The tubes were homogenised by vortexing so that they came into contact with the solution and centrifuged 3 times at 15,600 G for 5 min at 4 °C. This elution was divided into 4 aliquots of 100 μL, with only one being used for the test.

Before ELISA, the samples were diluted by mixing 10 µL of sample in 390 µL of RD5T buffer (0.025) as indicated in the protocol, thus obtaining a 40× dilution. This step was repeated twice and left at 80×.

In total, 40 samples (in duplicate) and 8 standards (in duplicate) were used, making a total of 96 wells.

The procedure began by adding 50 µL of the RD1-63 test diluent to each well. After this, 100 μL of standard or sample was added per well and covered with an opaque adhesive sticker for incubation for 2 h at room temperature in a plate shaker at 500 rpm. Afterwards, it was turned upside down and dried face down on paper. The process was repeated 3 times for 4 washes. Each wash was carried out with 400 µL of wash buffer (reference 895003 of the kit itself, Ref. HSLB00D). After the last wash, all of the wash buffer was removed by inverting the plate and drying on clean paper.

Once this was completed, 200 µL of human IL-1β HS conjugate was added to each well and covered again with a new adhesive sticker for incubation for 1 h at room temperature while shaking. Subsequently, a new wash was carried out. After that, 200 µL of Streptavidin Polymer-HRP (1×) was added to each well, and it was covered again with a new adhesive sticker for incubation for 30 min at room temperature on the plate shaker at 500 rpm. The washing was repeated, and 200 μL of substrate solution was added (100 μL of colour A + 100 μL of colour B) and incubated for 30 min at room temperature on the bench, well protected from light.

Finally, 50 μL of Stop Solution was added to each well and resuspended. The colour of the wells then changed from blue to yellow.

The results were read before 30 min at λ = 450 nm. To correct the absorbance, another reading at λ = 570 nm was taken due to possible imperfections in the plate. The reading was carried out on the Thermofisher MultiScan Go spectrophotometer.

### 2.8. Interpretation of Samples

The protocol that was followed was to take the average of the two readings of each sample or standard. To obtain more precise results, the average was subtracted from the absorbance that gave zero.

A corresponding working standard curve was created according to the absorbances obtained from the standards, representing the absorbances on the Y axis and the known concentration of the standards on the X axis. The X was removed from the equation of the straight line, and the calculation was carried out for each absorbance of the samples obtained.

Straight line: y = 0.1892x + 0.0295

If the samples had been diluted, the measured concentrations were multiplied by the dilution factor.

The minimum detectable dose was 0.033 pg/mL.

### 2.9. Statistical Analysis

The Kolmogorov–Smirnov test was applied to determine the normality of the numerical variables, concluding that, for the variables under analysis, in no case was the distribution normal.

To cross-check qualitative variables, the Chi^2^ test was carried out. To determine the groups that make the difference, Haberman’s corrected standardised residuals were used, which made it possible to obtain the significance of the cells independently. This significance implied that the percentage of the cell was statistically different from that corresponding to the total of the sample.

For the processing of categorical and numerical variables, the Mann–Whitney U test was applied as the variables under analysis did not have a normal distribution.

Given that the target variables followed a non-normal distribution, Sperman’s correlation was generally applied.

Statistical significance was indicated with the usual format (*p* < 0.05, *p* < 0.01, *p* < 0.001, *p* < 0.0001, and *p* < 0.00001); the lower the figure, the greater the significance.

## 3. Results

A study with a total of 96 implants was planned for 96 patients, of whom 54 were women and 42 were men.

### 3.1. Results According to Demographic Data

#### 3.1.1. Differences by Gender

There were no statistically significant differences in the degree of pain perceived by the female sex, classified on average as 2.14 ± 1.37, which was not very different from the degree of pain perceived by the male sex, classified as 1.90 ± 1.45.

However, with respect to inflammatory response by sex, greater differences were observed. At 24 h after placement of the rehabilitation, the degree of inflammation in women was 0.33 ± 0.82 compared to 0.05 ± 0.22 in men; at 48 h, the values were 0.44 ± 1.02 in women compared to 0.05 ± 0.22 in men; and on the day on which the inflammation subsided, the values were 1.89 ± 2.04 in women compared to 1.21 ± 0.98 in men.

IL-1β concentrations according to the degree of inflammation were 11.39 pg/mL for women and 7.12 pg/mL for men, without statistically significant differences (Table 1).

#### 3.1.2. Differences by Age

Regarding age, patients who were under 58 years old suffered less pain than those who were over 58 years old (Table 2).

IL-1β concentrations according to the degree of inflammation were 3.99 ± 30.51 pg/mL for patients younger than 58 years and 13.59 ± 33.54 pg/mL for those older than 58 years, with statistically significant differences (*p* < 0.05) (Table 3).

#### 3.1.3. Differences with Respect to Implant Location

Regarding the location of the implants, those placed in the mandible presented a higher degree of pain than those placed in the maxilla (Table 4).

IL-1β concentrations according to the degree of inflammation were 8.28 ± 33.77 pg/mL in the maxilla and 10.49 ± 29.88 pg/mL in the mandible, without reaching statistically significant differences.

### 3.2. According to Attachments Used

#### 3.2.1. Healing Cap or Healing Abutment

The pain and inflammatory response was evaluated based on the diameter of the healing abutment, which was always smaller than that of the rehabilitative abutment, in order to assess the soft-tissue response following the placement of the rehabilitative abutment.

Increased pain and inflammation were associated with healing abutments with smaller diameters (<4.86 mm) (Table 5).

IL-1β concentrations according to the degree of inflammation were 13.81 ± 33.86 pg/mL for abutments with diameters smaller than 4.86 millimetres and 4.17 ± 30.51 pg/mL for abutments with diameters larger than 4.86 millimetres (*p* < 0.01) (Table 6).

#### 3.2.2. Prosthetic Abutment

Finally, the pain and inflammatory response was observed based on the abutment diameter of the prosthetic abutment, which was always larger than the diameter of the healing abutment used previously.

When rehabilitated with a prosthetic abutment with a diameter less than 5.86 mm, the highest degree of pain occurred at the time of placement, with a value of 1.69 ± 1.22, while with a prosthetic abutment with a diameter greater than 5.86, the degree of pain was 2.39 ± 1.49. In this case, a statistically significant difference was observed (*p* < 0.05). For the rest of the times evaluated, i.e., at 24, 48, and 72 h, no statistically significant difference was observed.

The difference in concentrations of IL-1β according to the degree of inflammation between prosthetic abutments with diameters smaller and larger than 5.86 mm did not reach statistical significance.

On the other hand, with respect to the height of the prosthetic abutment, statistically significant differences were observed when comparing the degree of inflammation observed with respect to the use of abutments with diameters larger and smaller than 2.45 mm (Table 7).

The concentrations of IL-1β according to the degree of inflammation were 13.56 ± 34.72 pg/mL for prosthetic abutments with diameters less than 2.45 mm and 4.42 ± 29.60 pg/mL for abutments with diameters greater than 2.45, and they did not reach statistically significant differences.

## 4. Discussion

After analysis of the results, a greater inflammatory response was observed in the mandible than in the maxilla, both in the anterior and posterior sections. Inflammation causes cell migration to areas where there is greater oxygenation, directly affecting the gingival margin of the restorations [18]. For this reason, we decided to evaluate the existing inflammation margin and control it to avoid prolonged ischemia once the definitive prosthesis was installed.

The platform switching system described by Lazzara and Porter in 2006 based on the use of a narrower abutment in relation to the implant diameter [19] may reduce peri-implant bone resorption as it contributes to an improvement in inflammation control. It appears to help prevent peri-implant soft tissue recession over time compared to implants without platform switching [20,21], showing a positive effect on marginal bone levels compared to restorations without platform switching [22,23,24]. In this study, the platform change concept was applied to all implants.

Regarding the attachments used, healing abutments with diameters less than 4.86 mm showed greater pain and inflammation, with IL-1β concentrations reaching 13.81 ± 33.86 pg/mL, compared to 4.17 ± 30.51 pg/mL for abutments with diameters greater than 4.86 mm, with statistically significant differences between these diameters at *p* < 0.01.

On this point, there are studies that reveal the benefits of modifying the healing abutment in immediate surgeries by promoting the formation of stable and thicker peri-implant tissues [25]. Gamborena et al. (2015) assert that using abutments with a smaller custom diameter can provide the following advantages: support for connective tissue grafts in the most coronal position; improved papilla formation, allowing primary flap closure; support for an immediate provisional restoration or a restoration bonded to adjacent teeth, as is the case with Maryland bridges; and eliminating vertical loading of the grafted tissue during the healing phase [25]. In the long term, the use of narrower, customised abutments allows the thickness of the tissues initially operated on during the first surgery to be maintained [26].

When direct-to-implant prosthetic abutments are used, the probability of cervical-to-apical migration due to continuous gingival inflammation, in addition to the number of times the attachments are connected and disconnected, generates repeated changes, which does not occur with the use of a transepithelial abutment that avoids this constant manipulation of soft tissues [27,28], in addition to reducing the micro-space that may exist at the implant–abutment interface [27]. However, the use of conventional abutments shows a clinically acceptable microgap, as reported in the scientific literature [29,30,31,32].

It should be clarified that transepithelial abutments were not used in this study, and all abutments were exactly the same to avoid the risk of bias. Significant changes in pain were observed with the use of prosthetic abutments with platforms larger than 5.86 mm; thus, the larger the diameter of the prosthetic abutment, the greater the pain response, reaching levels of significance only on the day of prosthetic rehabilitation placement (*p* < 0.05). Both the prosthetic abutments and the definitive crowns were designed without altering the same work protocol in order to obtain the same result in all cases, even in cases where there may have been implants with angulations that could require corrections. Although there are studies indicating that the process of overlaying titanium custom abutments with pre-scan custom abutment library data improves the accuracy of the digital scan performed with respect to an intraoral scan, there was no risk of bias because all cases were scanned with the same scanning abutment [33,34]. Studies have shown that the use of customised abutments leads to an increase in the final abutment size, improved retention of the prosthetic work, and reduced angulation of the abutment in relation to the implant axis, thus reducing the risk of unscrewing or fracturing the dental screw. Therefore, the use of customised abutments provides stability to aesthetically compromised peri-implant tissues [35,36].

The final crowns were milled in the dental laboratory with multilayer zirconium oxide discs, complying with the cementing protocol for a cement–screw system, using dual-polymerisation self-adhesive cement in an indirect way, highlighting the importance of this technique involving the elimination of cement remains, which can cause constant inflammation in the gums. The crowns were finally installed in each of the 96 patients. There are studies that indicate that the cemented option is reliable, but others indicate that cemented restorations are associated with a higher rate of biological complications with respect to screw-retained restorations [37,38,39].

Studies on the level of IL-1β in crevicular fluid comparing cemented and screw-retained implant-supported rehabilitations have concluded that the clinical and radiographic parameters show no difference in the volume of peri-implant sulcular fluid and are thus comparable to each other in terms of clinical–radiographic status with IL-1β levels within the normal range [40]. In this study, all the restorations were screwed to implants, so there was no risk of bias in this respect. Under similar conditions, the level of IL-1β was found to be much higher in peri-implant crevicular fluid than in gingival crevicular fluid [41], so it is expected that in implant-supported rehabilitations, IL-1β values will be higher. Studies have indicated that painful and inflammatory processes are associated with increased levels of IL-1β. Higher levels of proinflammatory cytokines (interleukin (IL)-1β and IL-6) were observed in individuals with peri-implantitis compared to healthy implants [42,43,44]. In this study, similarly, the greater the pain and inflammatory response, the higher the IL-1β levels. This was especially the case when the healing abutment was less than 4.86 mm in diameter, with a pain grade of 0.73 ± 1.30, whereas with a healing abutment with a dimeter greater than 4.86, the pain grade was 0 ± 0. The statistically significant difference was *p* < 0.0001. Similarly, statistically significant differences with a *p*-value < 0.001 were found in the degree of inflammation between the use of healing abutments with diameters smaller and larger than 4.86 mm.

In summary, this study demonstrates practical implications for abutment selection in implant rehabilitation, especially in terms of controlling inflammation and postoperative pain. Application of the findings to a larger population can improve clinical outcomes in dental practice, highlighting the importance of considering the size and type of abutment to minimise inflammation and maximise patient comfort.

### Limitations

The present investigation is limited by the restricted temporal approach to sample collection for Il-1β, which was limited to a single 4 h post-rehabilitation interval. This limitation prevented the assessment of possible variations in parameters of interest over time, which could have provided a more complete and dynamic understanding of the effects of rehabilitation. Therefore, future research could benefit from a broader temporal approach encompassing multiple sampling points to enrich the understanding of the effects of rehabilitation in the context studied.

## 5. Conclusions

Greater inflammation was evident in cases in which healing abutments with reduced diameters, prosthetic abutments with larger diameters, and abutments with lower heights were used. The greater the degree of pain and inflammation, the higher the level of IL-1β. The length of the healing abutments must be longer than the initial gingival measurement to avoid future inflammation.

## Figures and Tables

**Table 1:** Medians of pain and inflammation and IL-1Β concentration with respect to sex at the time of placement (clinical).

Variable	Woman		Man	
	Average	D.E.	Average	D.E.
Degree of pain at the clinic	2.14	1.37	1.90	1.45
Degree of inflammation at the clinic	0.02	0.14	0.00	0.00
Concentration (pg/mL)	11.39	31.89	7.12	33

Data on the degree of pain and inflammation were obtained by conducting surveys. IL-1β concentration data were obtained by quantification using the ELISA technique. The Mann–Whitney U test was applied as the variables under analysis did not have a normal distribution. Statistical significance was established at *p* < 0.05. None of these results reached significance.

**Table 2:** Differences in the degree of pain and inflammation with respect to the age of the patients.

Variable	Up to 58 Years Old		Over 58 Years Old		Sign.
	Half	S.D.	Half	S.D.	
Degree of pain at the clinic	1.87	1.40	2.22	1.40	
Degree of pain at 24 h	0.24	1.00	0.50	0.96	<0.05
Degree of pain at 48 h	0.16	0.79	0.46	0.96	<0.05
Degree of pain at 72 h	0.12	0.59	0.20	0.54	
Degree of pain until the day of pain remission	0.24	1.00	0.76	1.52	<0.05
Day when the inflammation subsided	1.36	1.24	2.09	2.01	<0.05
Degree of inflammation at the clinic	0.00	0.00	0.02	0.15	
Degree of inflammation at 24 h	0.04	0.20	0.39	0.88	<0.05
Degree of inflammation at 48 h	0.04	0.20	0.52	1.09	<0.05
Degree of inflammation at 72 h	0.06	0.42	0.33	0.87	<0.05
Degree of pain until the day of inflammation remission	0.14	0.76	0.85	1.79	<0.05
Day when the inflammation subsided	1.18	0.90	2.04	2.18	<0.05

Data on the degree of pain and inflammation were obtained by conducting surveys. The Mann–Whitney U test was applied as the variables under analysis did not have a normal distribution. Statistical significance was established at *p* < 0.05. None of these results reached significance.

**Table 3:** Medians of pain and inflammation and IL-1Β concentration with respect to age at the time of placement (clinical).

Variable	Up to 58 Years Old		Over 58 Years Old		Significance
	Average	D.E.	Average	D.E.	
Degree of pain at the clinic	1.87	1.40	2.22	1.40	
Degree of inflammation at the clinic	0.00	0.00	0.02	0.15	
Concentration (pg/mL)	3.99	30.51	13.59	33.74	<0.05

Data on the degree of pain and inflammation were obtained by conducting surveys. IL-1β concentration data were obtained by quantification using the ELISA technique. The Mann–Whitney U test was applied as the variables under analysis did not have a normal distribution. Statistical significance was established at *p* < 0.05. None of these results reached significance.

**Table 4:** Differences in the degree of pain and inflammation with respect to the location of the implants.

Variable	Maxilla		Mandible		Sign.
	Half	S.D.	Half	S.D.	
Degree of pain at the clinic	1.85	1.34	2.12	1.43	
Degree of pain at 24 h	0.03	0.18	0.52	1.16	<0.05
Degree of pain at 48 h	0.00	0.00	0.45	1.05	<0.05
Degree of pain at 72 h	0.00	0.00	0.23	0.68	
Degree of pain until the day of pain remission	0.03	0.18	0.71	1.53	<0.05
Day when the inflammation subsided	1.13	0.72	1.98	1.93	<0.05
Degree of inflammation at the clinic	0.03	0.18	0.00	0.00	
Degree of inflammation at 24 h	0.00	0.00	0.31	0.77	<0.05
Degree of inflammation at 48 h	0.00	0.00	0.40	0.95	<0.05
Degree of inflammation at 72 h	0.00	0.00	0.28	0.82	quasi
Degree of pain until the day of inflammation remission	0.00	0.00	0.71	1.65	<0.05
Day when the pain subsided	1.00	0.00	1.88	2.00	<0.05

Data on the degree of pain and inflammation were obtained by conducting surveys. The Mann–Whitney U test was applied as the variables under analysis did not have a normal distribution. Statistical significance was established at *p* < 0.05. None of these results reached significance.

**Table 5:** Differences in pain and inflammation with respect to the diameter of the healing cap.

Variable	Up to 4.86 mm		More than 4.86 mm		Sign.
	Half	S.D.	Half	S.D.	
Degree of pain at the clinic	2.46	1.22	1.61	1.45	<0.01
Degree of pain at 24 h	0.73	1.30	0.00	0.00	<0.0001
Degree of pain at 48 h	0.60	1.18	0.00	0.00	<0.001
Degree of pain at 72 h	0.31	0.78	0.00	0.00	<0.01
Degree of pain until the day of pain remission	0.98	1.71	0.00	0.00	<0.0001
Day when the pain subsided	1.13	0.72	1.98	1.93	<0.0001
Degree of inflammation at the clinic	0.02	0.14	0.00	0.00	
Degree of inflammation at 24 h	0.42	0.87	0.00	0.00	<0.001
Degree of inflammation at 48 h	0.54	1.07	0.00	0.00	<0.001
Degree of inflammation at 72 h	0.38	0.94	0.00	0.00	<0.01
Degree of pain until the day of inflammation remission	0.96	1.86	0.00	0.00	<0.001
Day when the inflammation subsided	2.19	2.25	1.00	0.00	<0.001

Data on the degree of pain and inflammation were obtained by conducting surveys. The Mann–Whitney U test was applied as the variables under analysis did not have a normal distribution. Statistical significance was established at *p* < 0.05. None of these results reached significance.

**Table 6:** Medians of pain and inflammation and IL-1Β concentration with respect to scar plug diameter.

Variable on Pain and Inflammation According to Healing Abutment (Diameter)	Up to 4.86 mm		Over 4.86 mm		Sign.
	Half	S.D.	Half	S.D.	
Degree of pain at the clinic	2.46	1.22	1.61	1.45	<0.01
Degree of pain at 48 h	0.60	1.18	0.00	0.00	<0.001
Degree of inflammation at the clinic	0.02	0.14	0.00	0.00	
Degree of inflammation at 48 h	0.54	1.07	0.00	0.00	<0.001
Concentration (pg/mL)	13.81	33.86	4.17	30.51	<0.01

Data on the degree of pain and inflammation were obtained by conducting surveys. IL-1β concentration data were obtained by quantification using the ELISA technique. The Mann–Whitney U test was applied as the variables under analysis did not have a normal distribution. Statistical significance was established at *p* < 0.05. None of these results reached significance.

**Table 7:** Differences in pain and inflammation with respect to prosthetic abutment height.

Variable	Up to 2.45 mm		More than 2.45 mm		Sign.
	Half	S.D.	Half	S.D.	
Degree of pain at the clinic	1.99	1.35	2.08	1.46	
Degree of pain at 24 h	0.48	1.09	0.25	0.86	
Degree of pain at 48 h	0.46	1.01	0.15	0.71	<0.05
Degree of pain at 72 h	0.21	0.62	0.10	0.52	
Degree of pain until the day of pain remission	0.69	1.50	0.29	1.03	
Day when the pain subsided	1.85	1.83	1.56	1.53	
Degree of inflammation at the clinic	0.00	0.00	0.02	0.14	
Degree of inflammation at 24 h	0.33	0.78	0.08	0.45	<0.05
Degree of inflammation at 48 h	0.46	1.01	0.08	0.45	<0.05
Degree of inflammation at 72 h	0.33	0.91	0.04	0.29	<0.05
Degree of pain until the day of inflammation remission	0.81	1.76	0.15	0.77	<0.05
Day when the inflammation subsided	1.98	2.10	1.21	1.03	<0.05

Data on the degree of pain and inflammation were obtained by conducting surveys. The Mann–Whitney U test was applied as the variables under analysis did not have a normal distribution. Statistical significance was established at *p* < 0.05. None of these results reached significance.

## Data Availability

The data presented in this study are available on request from the corresponding author.
